# Supplementary material for: What are the spatio-temporal differentiation characteristics and driving factors of the coupling coordination degree between green finance and ecological efficiency? Evidence from 84 cities in western China
Source: PLoS One. 2025 Oct 30;20(10):e0334001. doi: 10.1371/journal.pone.0334001 (PMC12574906; doi:10.1371/journal.pone.0334001)
Supplement: Attachment — Submitted filename: Response to Reviewers.docx [file pone.0334001.s002.docx]

**Response to Reviewers**

Dear Editor,

Thank you for arranging the review and the reviewer's valuable comments. We have read your manuscript in detail and submitted the **Response to Reviewers** and the **Revised Manuscript with Track Changes** and the **Manuscript**. In addition, since we have modified the pictures in the original text, we have uploaded a new file called **"Modified Pictures".** According to the two reviewers' comments. All the changes have been marked. Thanks again to the editors and reviewers for their help.The following are the responses to the editor and reviewers:

2. We note that the grant information you provided in the‘Funding Information’and‘Financial Disclosure’sections do not match.

Responds: Thank you for your careful review.The following are the 'Funding Information' and 'Financial Disclosure' of this article.

Funding

This research was financially supported by the National Social Science Foundation “Research on uncoordinated coupling identification and coordinated development path of green finance and ecological resources in western China” (No. 21CJY033)

3. When completing the data availability statement of the submission form, you indicated that you will make your data available on acceptance. We strongly recommend all authors decide on a data sharing plan before acceptance, as the

Responds: Thank you for your careful review.If necessary, we can provide relevant data.

4. We require you to either (1) present written permission from the copyright holder to publish these figures specifically under the CC BY 4.0 license, or (2) remove the figures from your submission:

Responds: Thank you for your careful review.We have deleted the original Figures of 1, 5-1, 5-2, 5-3, 5-4, 7-1,7- 2, 7-3, 7-4, 10-1, 10-2, 10-3 and 10-4, replace with other images. Please refer to Figures 4, 6, 9 and 10 for details.

**The following are the responses to Reviewer 1 and Reviewer 2.**

Response to Reviewer 1 Comments

Dear reviewer:

We are very grateful to your comments for the manuscript. All your suggestions have important guiding significance for our paper writing and scientific research. According with your advice, we amended the relevant part in manuscript. The answers to your questions are as follows.（Please refer to the attachment for detailed reply.）

1.It is suggest to write questions at the end of introduction, and write what methods/approaches you will use to answers of those study questions.
Responds: Thanks to your careful review, at the end of the introduction, we added the problems to be solved and the solutions.:

Line 113-130:Based on the above background, this study intends to address the following core issues: What is the current status of the coupling and coordination between green finance and the ecological environment system in western cities? What are the characteristics of spatio-temporal differentiation? What are the key factors driving the coupled and coordinated development of the two systems? How can the level of coordinated development of green finance and ecological environment in western cities be enhanced through institutional innovation and policy optimization? To better study the coordinated development of green finance and ecological efficiency in the western region of China, it is necessary to apply the systems view and collaborative thinking. This paper will construct the index system of the two systems, measure their coupling coordination level by using the coupling coordination degree model, and conduct spatio-temporal evolution analysis by using methods such as spatial econometric analysis. Finally, the spatial econometric model will be used to explore its influencing factors. To promote the coupled and coordinated development of the two systems, targeted policies are proposed.

2.The fourth point of innovation in the paper does not seem to be an innovation point, but rather a research significance.

Responds: Thanks to your careful review, This article has deleted innovation point 4.

Line276-280:~~(4) The driving factors affecting CCD-GFEE are determined using the spatial econometric model. This offers a solid scientific foundation for developing policies that support ecological environmental protection and green economic development.~~

3. What are the selection principles for the 84 cities in the research sample?

Responds: Thanks to your careful review, The selection of 84 cities was mainly based on considerations of data completeness and accuracy, and relevant explanations were provided in the article:

Line274-285：It should be noted here that due to the influence of data collection channels, in the key data sources, namely the "China Urban Statistical Yearbook (2007-2021)" and the "China Urban Construction Statistical Yearbook (2007-2021)", the data for many indicators of cities such as Lhasa, Xigaze, Chamdo, and Nagqu in Tibet, Haidong City and Haibei Tibetan Autonomous Prefecture in Qinghai, and Hami City and Altay in Xinjiang are severely lacking. Based on the principles of data integrity and operability, these cities with severely missing data were excluded from the evaluation in this study. Ultimately, 84 cities with relatively complete and representative data were selected as the research samples, accounting for over 60% of all cities in the western region and covering the majority of cities in the western region.

4.Many formulas, letters in the paper are not neat and need to be standardized.

Responds: Thanks to your careful review,We have modified the formula and letters,Specifically as follows:

Line311-314:(1) Data standardization.

$$Mij=\frac{X_{ij}-\min\left(X_{ij}\right)}{\max\left(X_{ij}\right)-\min\left(X_{ij}\right)}+0.001$$

$$Mij=\frac{\max\left(X_{ij}\right)-X_{ij}}{\max\left(X_{ij}\right)-\min\left(X_{ij}\right)}+0.001$$

Line358:**3.3.3 Coupling coordination degree(CCD) model**

5.The clarity of the images in the text is low and needs to be modified.

Responds: Thanks to your careful review, We have modified the unclear pictures in the article.The following is the clear picture after modification:

|   （a） | （b） |
| --- | --- |

Fig4. Spatial evolution trend of green finance index

|   （a） |   （b） |
| --- | --- |

Fig6. Spatial evolution of urban EE in western China

Fig8. Mean value of CCD-GFEE of cities in the western region

|  |  |
| --- | --- |
|  |  |

Fig9. Distribution of CCD-GFEE in cities in the western region

|   (a) |   (b) |
| --- | --- |

Fig10. Spatial distribution of CCD-GFEE in cities in the western region

6. The font formats in Table 1 are not consistent.

Responds: Thanks to your careful review, We have modified the font format in Table 1.

Line501：

Table 1. Ecological efficiency index system

| Indicator Type | Category | Indicator description | Unit |
| --- | --- | --- | --- |
| Input index | Capital | Total investment in fixed assets | 10 thousand yuan |
|  | Labor force | Employment number | Person |
|  | Resources | Total water supply | 10 thousand cubic meters |
|  |  | Use of liquefied petroleum gas | Ton |
|  | Public service | Number of schools | Institute |
|  |  | quantity of beds in  hospitals and health centers | Unit |
| Expected output | Urban economic development level | GDP | 100 million yuan |
|  | Urban greening | Green land rate in built-up area | % |
| Undesirable output | Waste water Discharge | Industrial waste water discharge | 10 thousand tons |
|  | Exhaust emission | Industrial sulfur dioxide emissions | Ton |

7. Table 6 does not use a three line table and is not consistent with the previous table format.
   Responds: Thanks to your careful review, We have modified Table 6.The specific modifications are as follows:

Table 6. Estimate and test results of spatial econometric model (spatial fixed effect model)

| variable | $SAR$ | $SEM$ |
| --- | --- | --- |
| ER | 0.0071  ( 0.9113) | 0.0058  ( 0.7458) |
| PS | -0.0080***  ( -3.5781) | -0.0088***  (-3.9466) |
| IS | 0.1095***  (6.0375) | 0.0713***  (3.7201 ) |
| EL | 0.0335***  ( 7.3760) | 0.0271***  (6.3141) |
| TP | 0.0112***  ( 4.8925) | 0.0109***  (4.7640) |
| GI | -0.0325*  (-1.7271) | -0.0271  (-1.3965) |
| $W*dep. \mathrm{var}.$ | -0.2360***  ( -3.9009 ) |  |
| $spat. aut .$ |  | 0.2070***  (3.6817) |
| $R-squared$ | 0.7378 | 0.7442 |

8.Why did the spatial econometric model be selected to measure the influencing factors of coupling coordination degree, rather than other methods? This is not clearly stated in the paper.
Responds: Thanks to your careful review, this article further explains the reasons for choosing the spatial econometric model to measure the influencing factors of CCD.

Line678-685:

Based on the results of the global Moran index mentioned earlier (see Table 4), CCD-GFEE has significant spatial correlation in space. That is, the coupling coordination degree and its influencing factors do not exist independently in space but are spatially correlated. Moreover, the research data are urban panel data with spatial attributes. Traditional methods may lead to result bias due to ignoring spatial effects. The spatial econometric model can capture spatial correlations and correct biases, and thus is more suitable for measuring the influencing factors of CCD-GFEE.

9.The citation format for references is inconsistent, with some citing the first letter of the name and others not.

Responds: Thanks to your careful review, We have made modifications based on the latest article literature insertion format of the journal, for example:

Line146-147:Zhao, Li [4] sees GF as a financing tool to aid in efforts for sustainable development and environmental preservation.

4.	Zhao L-T, Li F-R, Wang D-S. How to achieve the common developments of green finance and clean energy in China? Evidence from coupling coordination evaluation. Ecological Indicators. 2023;155. doi: 10.1016/j.ecolind.2023.111011.

Line-189-192:Xiao, Wang [35] presented a two-stage network DEA framework integrating the segments of the government and industry and assessed the EE of 84 resource-based communities in the years following the financial crisis.

35.	Xiao H, Wang D, Qi Y, Shao S, Zhou Y, Shan Y. The governance-production nexus of eco-efficiency in Chinese resource-based cities: A two-stage network DEA approach. Energy Economics. 2021;101:105408. doi: <https://doi.org/10.1016/j.eneco.2021.105408>.

Response to Reviewer 2 Comments

Dear reviewer:

We are very grateful to your comments for the manuscript. All your suggestions have important guiding significance for our paper writing and scientific research. According with your advice, we amended the relevant part in manuscript. The answers to your questions are as follows.（Please refer to the attachment for detailed reply.）

（1）The logic in section 3.3.1 lacks clarity. The steps of the entropy weight method are not sequentially labeled from the first step. Additionally, there are two sections numbered 3.1, which needs correction.
   Responds: Thanks to your careful review, We marked the entropy weight method from the first step and modified the sections numbers.

Line311:

(1) Data standardization.

$$Mij=\frac{X_{ij}-\min\left(X_{ij}\right)}{\max\left(X_{ij}\right)-\min\left(X_{ij}\right)}+0.001$$

$$Mij=\frac{\max\left(X_{ij}\right)-X_{ij}}{\max\left(X_{ij}\right)-\min\left(X_{ij}\right)}+0.001$$

(2) The paper’s readability could be improved. It is recommended to insert tables and figures within the text to enhance readability and facilitate review. Furthermore, the current images are blurry and need to be replaced with higher-quality versions.

Responds:Thank you for your careful review. According to the submission requirements of this journal, please attach the pictures. The insertion positions of each picture have been shown in the text.We have revised the chart to make it clearer. The specific content is as follows:

|   （a） | （b） |
| --- | --- |

Fig4. Spatial evolution trend of green finance index

|   （a） |   （b） |
| --- | --- |

Fig6. Spatial evolution of urban EE in western China

Fig8. Mean value of CCD-GFEE of cities in the western region

|  |  |
| --- | --- |
|  |  |

Fig9. Distribution of CCD-GFEE in cities in the western region

|   (a) |   (b) |
| --- | --- |

Fig10. Spatial distribution of CCD-GFEE in cities in the western region

(3) On line 495, the meaning of “NOTE” is unclear, and this annotation is not properly identified within the text.
    Responds: Thanks to your careful review, This annotation explains exactly what the six high energy-consuming industries include and adds the content of this annotation to the original green indicator system section.

Line435-440: (The six industries with high energy consumption include chemical raw materials and chemical products manufacturing industry, non-metallic mineral products industry, petroleum, coal and other fuel processing industry, electric power, heat and gas production and supply industry, the industry of rolling and smelting ferrous metal, the non-ferrous metal rolling and smelting sector.)

(4) Some section titles do not accurately reflect their content. For instance, in section 3.4.3, the title “Driver index construction of CCD-GFEE” does not match the content, which states, “This section adopts spatial econometric modeling and other methods to further explore the driving elements behind the coordinated development of the two.”

Responds: Thanks to your careful review, this part of the content is the construction of the CCD-GFEE driver factor index system and modifies the title of 3.4.3.

Line481:3.4.3 Construction of CCD-GFEE Driver Factor Index System

(5) The analysis of calculation results lacks depth. It primarily focuses on surface-level data interpretation without delving into specific underlying causes.

Responds: Thanks to your careful review, We have added a specific analysis of the reasons in the fifth part of the article.See lines 779-905 for details.Specifically as follows:

(1) On the whole, green finance generally shows a low level. This might be due to the fact that the number of financial institutions in the western region is small and their distribution is sparse, and the local fiscal and enterprise financial strength is limited, making it difficult for them to bear the high initial investment and long payback period of green projects. In addition, the western region is an ecologically fragile area in China. However, it has long relied on resource-based industries to drive its economy. The green transformation of industries requires high costs. In the short term, it is more inclined to maintain the stability of traditional industries, resulting in insufficient policy implementation and resource input for GF support. From the standpoint of the evolution of time, The GF level primarily exhibits an increased trend between 2007 and 2021, which agrees with the research done by Yi, Hao [80]，and the southwest region is growing more quickly than the northwest region. From the standpoint of the evolution of space, the overall development level of urban GF in western China is still rather low, with large regional differences, the study of Yuan, Jia [81] also shows that GF shows obvious regional differences. In addition to the high level of GF development in some cities, the level of GF in other regions is always low, which indicates that most cities in western China have plenty of space for improvement. In space, The northwest area of the high level city has gradually given way to the southwest area . The southwest region enjoys the support of national strategies such as the Chengdu-Chongqing Twin-City Economic Circle and Kunming as a regional international center city, making its policy dividends more concentrated. The overall economy in the northwest region is fragmented and regional coordination is insufficient, making it difficult to promote the development of GF. In addition, the industrial structure in the southwest region is greener, while that in the northwest region relies on high-carbon industries and has a prominent feature of heavy industrial structure.

（2）On the whole, the urban ecological efficiency shows a relatively high level, but it is still not DEA effective. As shown by Xue, Yue [5], the EE in the western region is low. This may be due to the fact that the public service input is included in the index system in this paper, which leads to the difference while determining the efficiency value. From the standpoint of the evolution of time, the overall average efficiency shows a slightly fluctuating upward trend, This is also confirmed by the research of [82]. The highest value of ecological efficiency appeared in 2017, however, the urban ecological development in the southwest region is faster than that in the northwest region. which agrees with the research done by [5]. Northwest China leads in efficiency values thanks to the short-term efficiency advantage of resource-based industries, but ecological fragility and transformation pressure have limited its development speed. Although the short-term efficiency in Southwest China is relatively low due to high ecological investment, the synergy of natural conditions, policy support and industrial transformation has accelerated the pace of ecological development. From a spatial standpoint, the western region of China has a higher level of urban performance as a whole, and the variations amongst regions are substantial. Southwest area efficiency is lower than northwest region efficiency. which agrees with the research done by Xue, Yue [5] , The cities with low EE are mainly located in Guangxi, Guizhou and Gansu provinces. This is similar to the study of Xue, Yue [5]. The spatial difference of EE between cities in Guangxi Province and Gansu Province is large, which indicates that the EE of cities in Guangxi and Gansu province is not balanced.

(3) From the standpoint of the evolution of time, the CCD-GFEE of cities in western China is mainly distributed between 0.4-0.5, with little overall change and a slight increase trend. This might be due to the lagging development of GF, which has limited support capacity for EE. Meanwhile, the "low starting point and slow improvement" of EE in the western regions have restricted the coordinated development with GF. This is different from the research of Zhang [83], This may be due to the fact that the study's scope is different from the prefecture-level city in this study. From the standpoint of the evolution of time, the CCD-GFEE's spatial correlation is positive, showing obvious aggregation effect, which agrees with the research done by Zhang, Geng [60], This might be due to the lagging development of GF, which has limited support capacity for EE. Meanwhile, the "low starting point and slow improvement" of EE in the western regions have restricted the coordinated development with GF. The spatial aggregation effect of CCD-GFEE first went up, then down. This might be because as the development stage advanced, the internal differentiation within the region intensified, breaking the initial synchronicity. Significantly improved coupling coordination was evident in the western region's CCD-GFEE spatial distribution, but the CCD was still at a low level, and the regional differentiation was obvious, Zhang, Geng [60] came to a similar conclusion. This might be due to the disparity in resources, policies and industries between core cities and medium and small-sized cities, which has exacerbated regional differentiation. Inner Mongolia and the provinces of Shaanxi and Sichuan are home to the majority of the cities with high CCD-GFEE. The CCD of Chengdu city is the highest, which is in the stage of almost coordination. The CCD in northwest China is higher than that in Southwest China, this might be due to the fact that the resource-based industries in the northwest region initiated a systematic green transformation earlier. Moreover, relying on its advantages in wind and solar energy resources, the northwest region has formed a complete new energy industrial chain. Additionally, the governance of ecologically fragile areas in the northwest region is more urgent, and policy and financial input are more precise. The cities with low CCD-GFEE in western China are decreasing, while the cities with high CCD-GFEE are increasing. Most cities are mainly distributed in the L5 level, that is, the Barely coordination stage. The CCD-GFEE of Xi 'an, Chengdu and Chongqing have reached the Intermediate coordination stage, and all of these cities serve as either national central capitals or provincial capitals. The cities where the CCD-GFEE is always low are Baiyin, Panzhihua and Jiayuguan, and Chongqing has achieved the largest phased leap-over.

（4）Viewed through the lens of CCD-GFEE driving factors, environmental regulations' effects on CCD-GFEE is not significant, the economic development level in the western region is relatively lagging behind. To ensure employment and short-term economic growth, local governments may have a tendency towards "lenient" environmental regulations. The constraints imposed by environmental regulations on enterprises' polluting behaviors are insufficient, making it difficult to force enterprises to enhance EE through green technological innovation. Meanwhile, due to the lack of clear regulatory guidance and incentives, green financial tools are also difficult to form synergy with environmental regulations, resulting in an insignificant promoting effect on the degree of coupling and coordination. While the impact of population size and government intervention on CCD-GFEE is negative, The ecological environment in the western region is fragile and its environmental carrying capacity is limited. The expansion of population size will directly increase resource consumption and pollution emissions, leading to a decline in EE. The results of Zhang, Geng [60] are close to this. However, different from the research conclusion of Zhang [83], This may be due to different government industrial policies in different regions. The influence of industrial structure, economic level and technological progress on CCD-GFEE is positive. The optimization of industrial structure is often accompanied by the agglomeration of green industries. Such agglomeration can not only reduce the ecological cost per unit output through the scale effect, but also attract the concentrated investment of green financial resources, there by increasing the CCD-GFEE. The improvement of economic level lays the foundation for the coordinated development of GF and EE by enhancing the capacity of resource supply and optimizing development goals. The positive impact of technological progress on the degree of coupling coordination is reflected in not only directly enhancing EE but also providing application scenarios and risk control tools for GF, achieving a deep integration of the two, Zhang, Geng [60] came to a similar conclusion.
